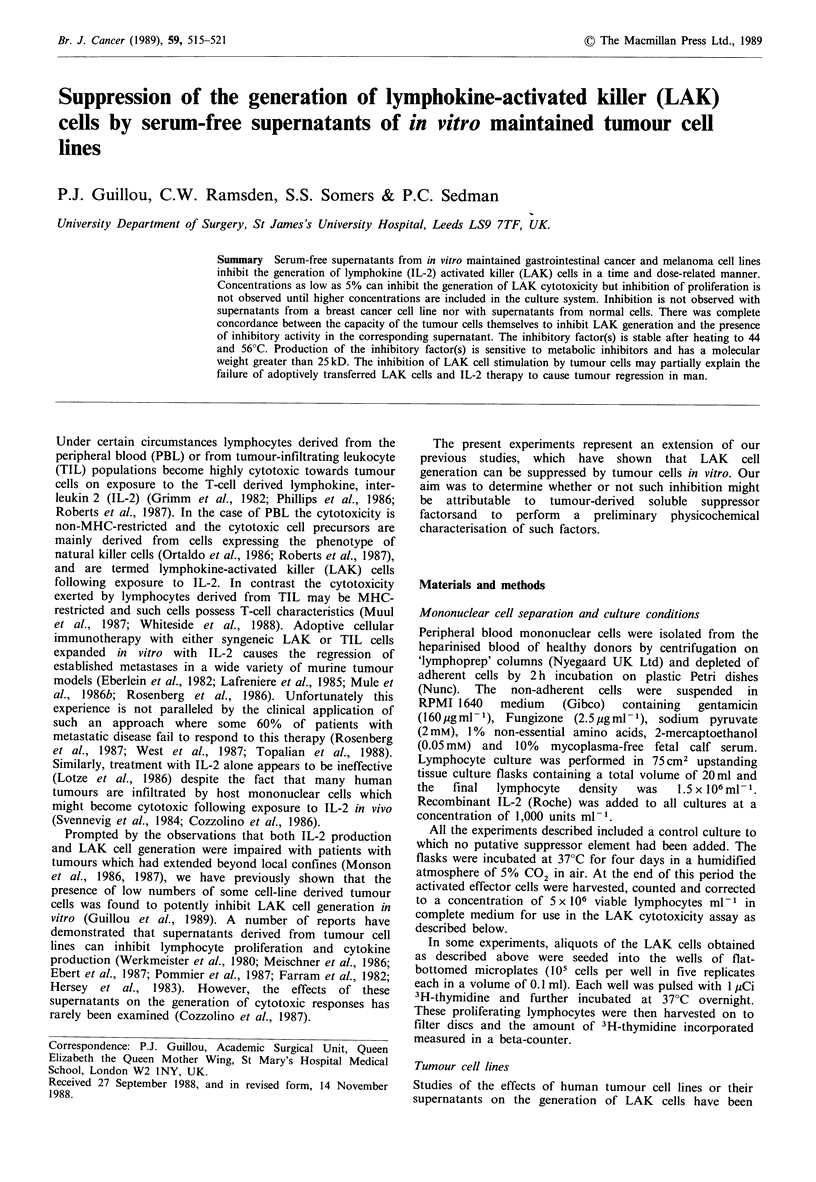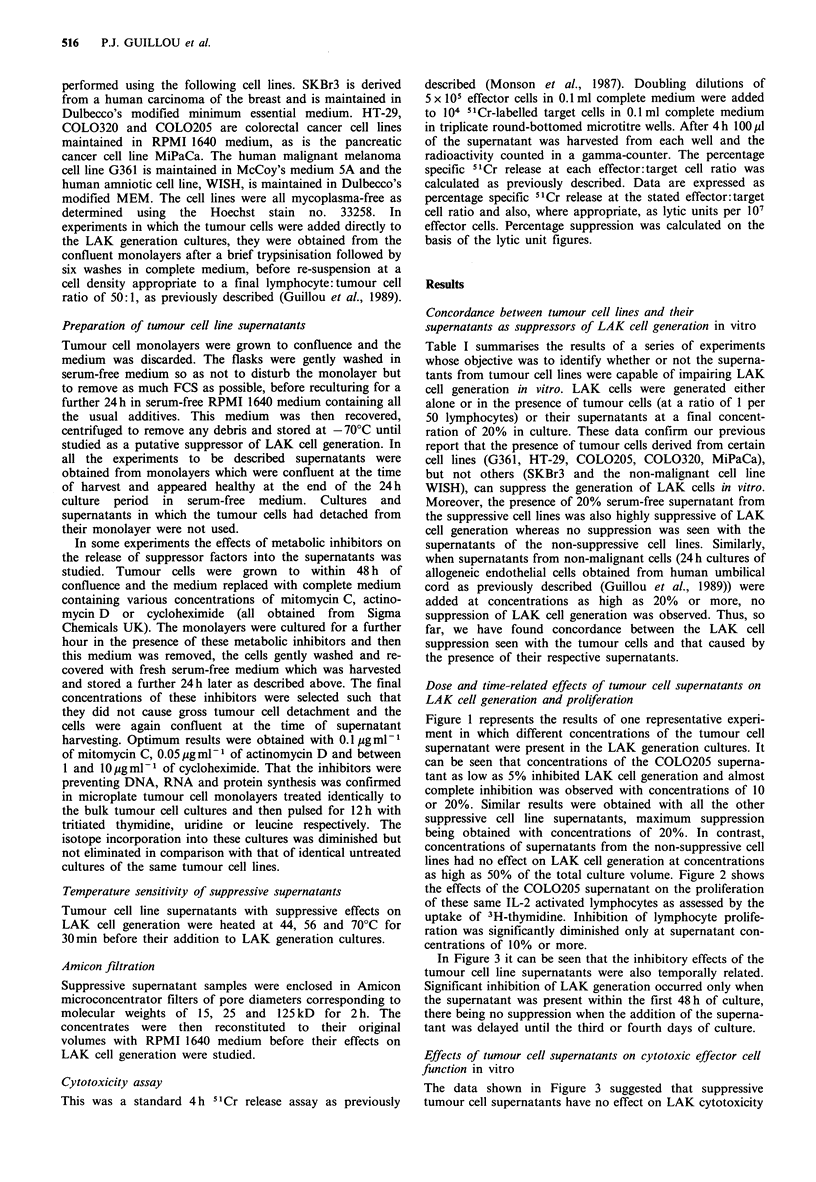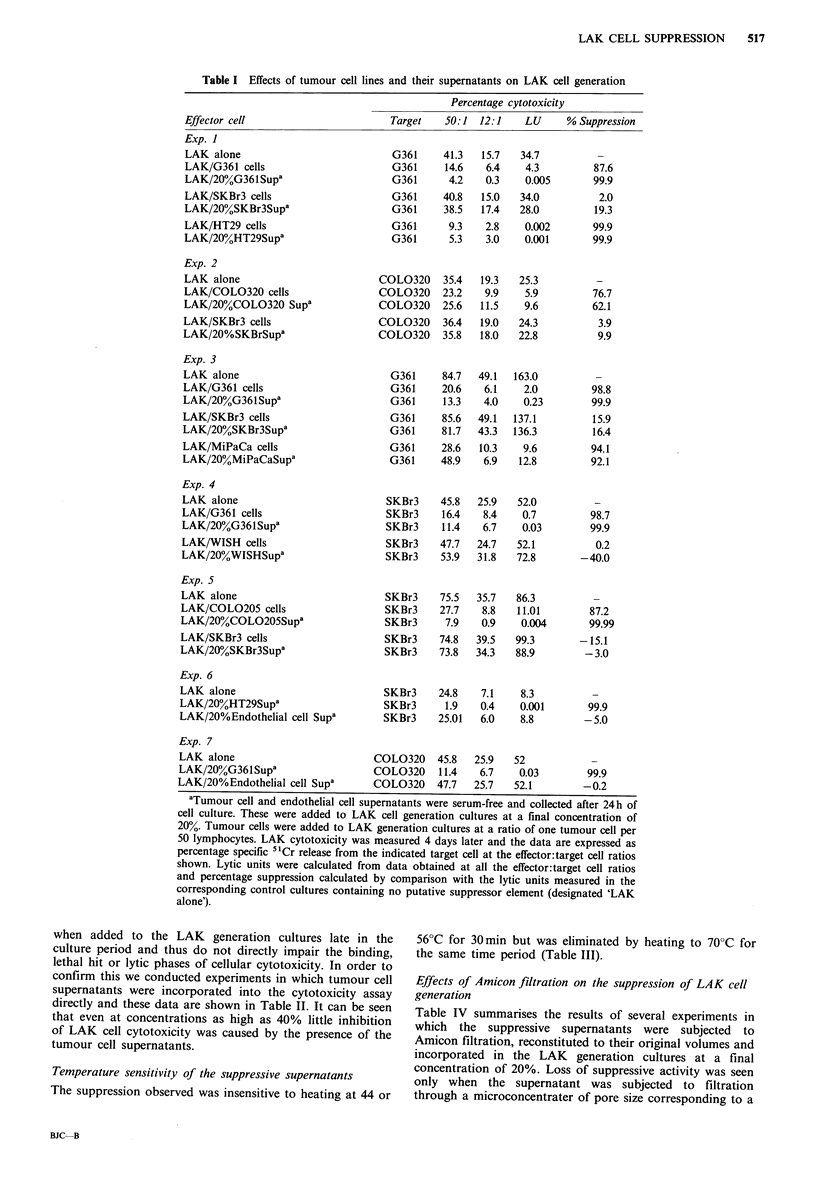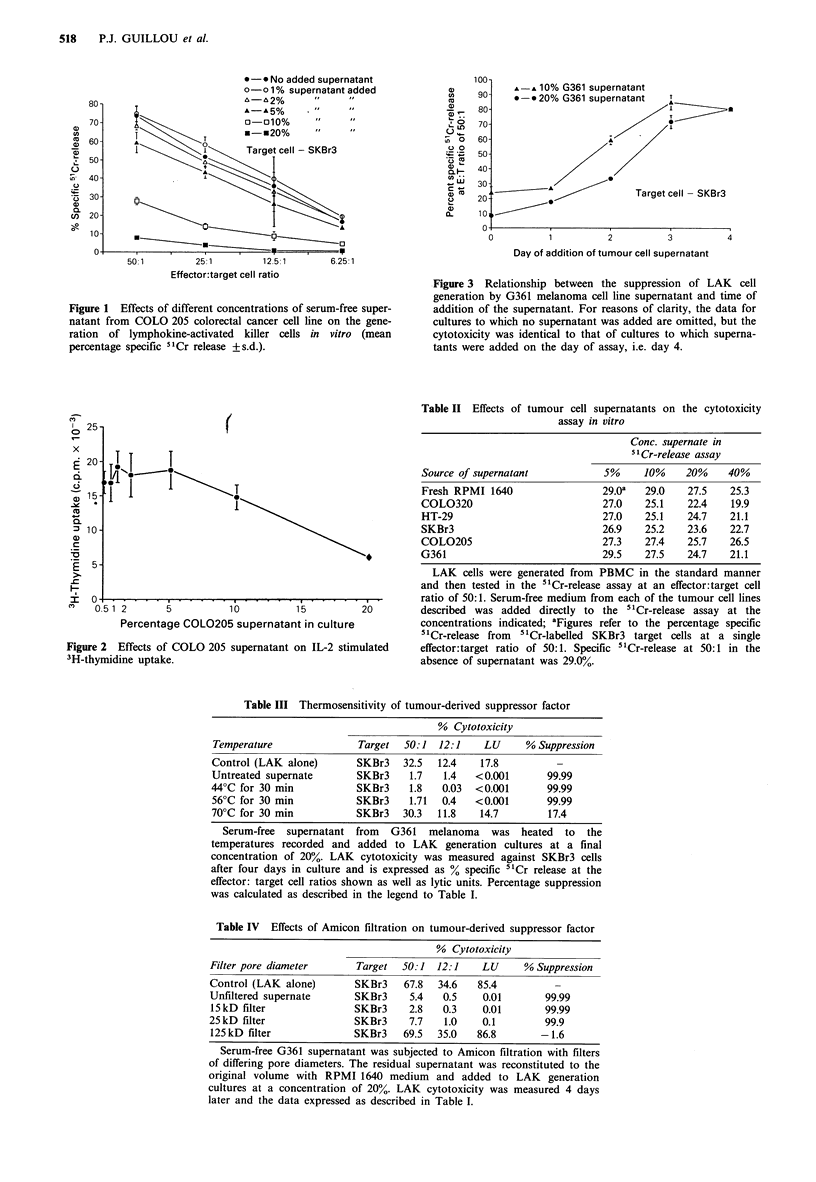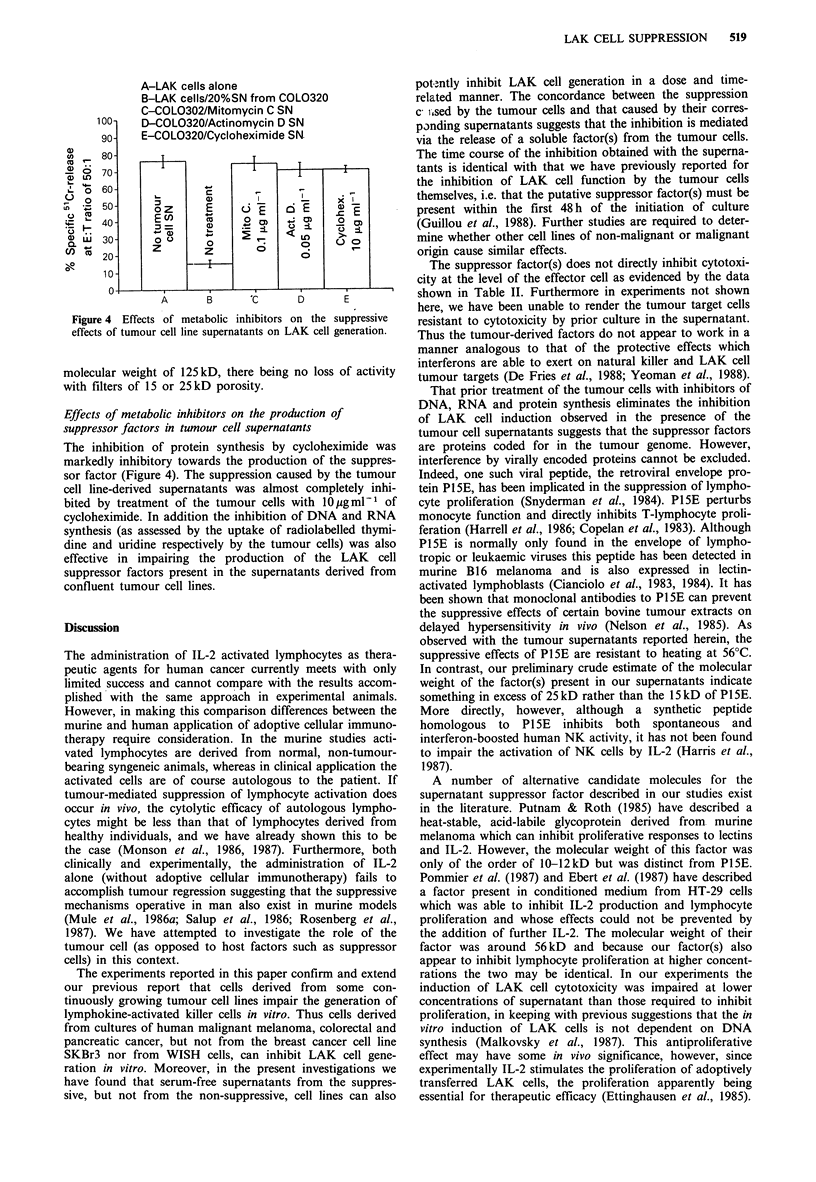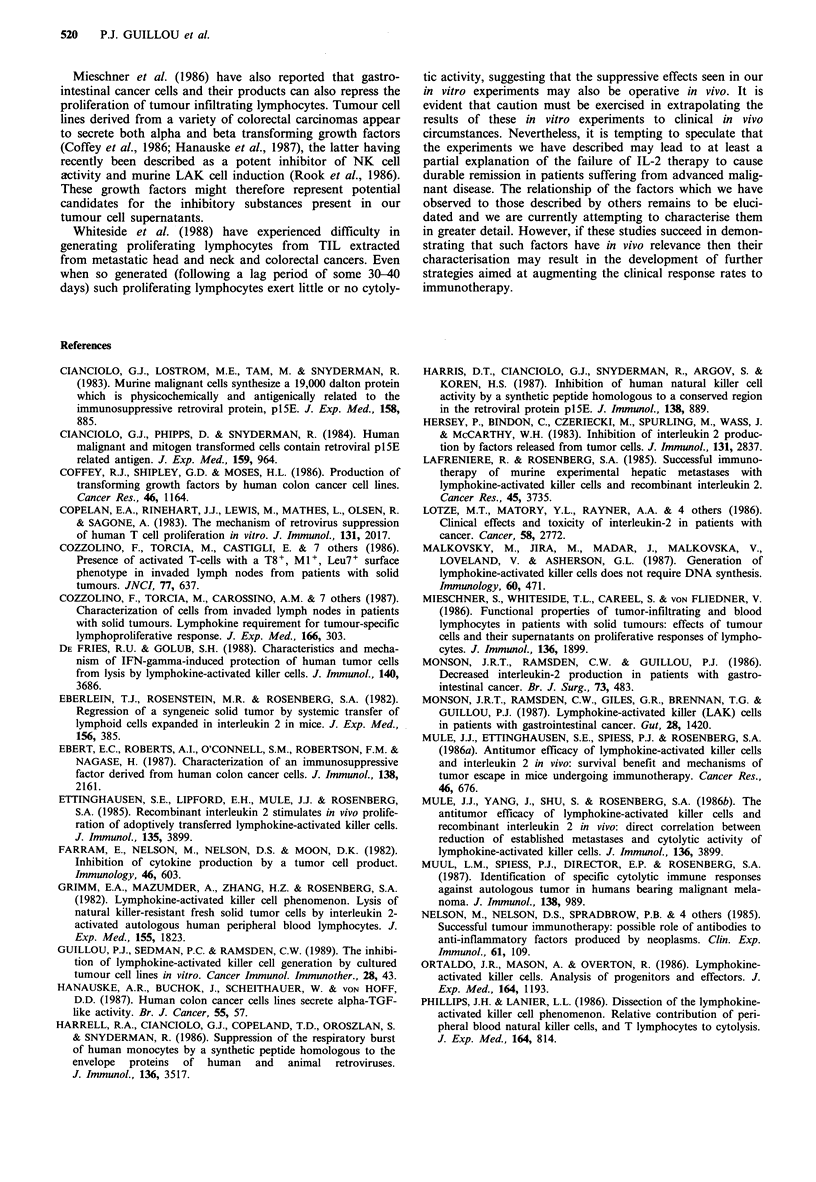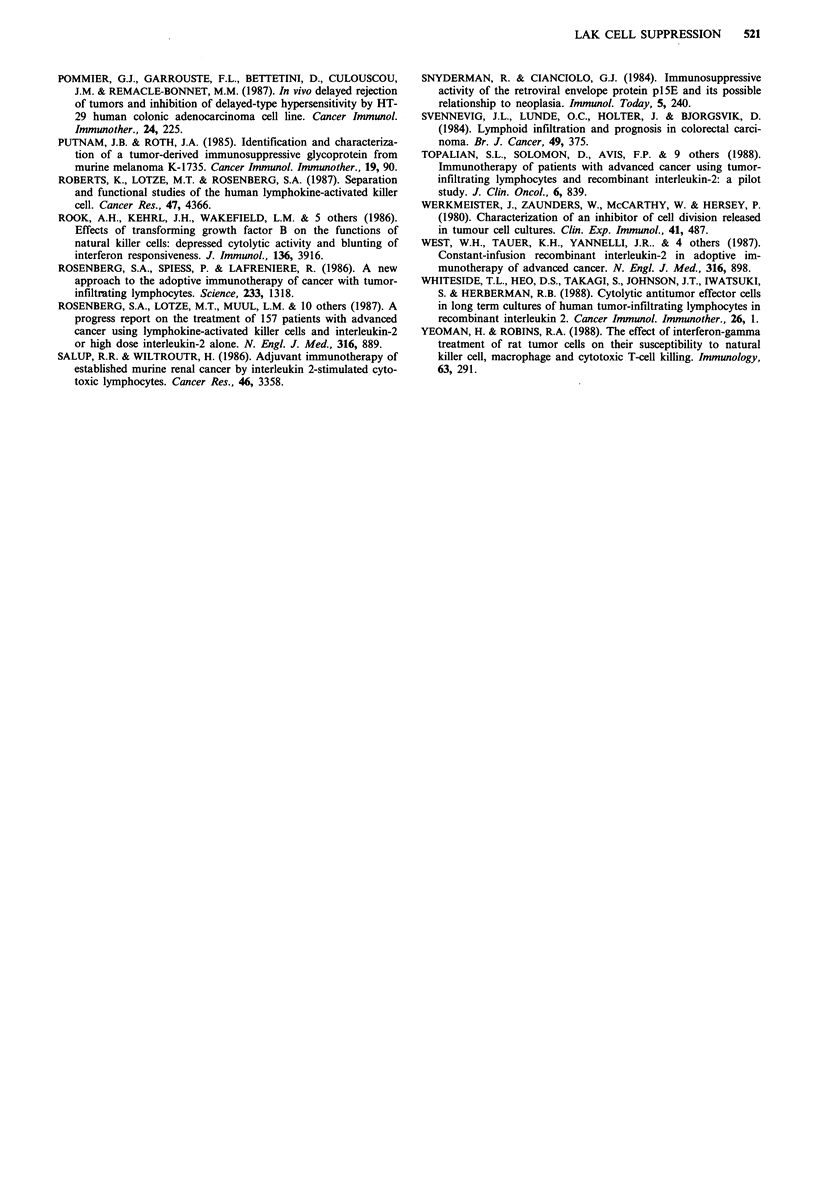# Suppression of the generation of lymphokine-activated killer (LAK) cells by serum-free supernatants of in vitro maintained tumour cell lines.

**DOI:** 10.1038/bjc.1989.106

**Published:** 1989-04

**Authors:** P. J. Guillou, C. W. Ramsden, S. S. Somers, P. C. Sedman

**Affiliations:** University Department of Surgery, St James's University Hospital, Leeds, UK.

## Abstract

Serum-free supernatants from in vitro maintained gastrointestinal cancer and melanoma cell lines inhibit the generation of lymphokine (IL-2) activated killer (LAK) cells in a time and dose-related manner. Concentrations as low as 5% can inhibit the generation of LAK cytotoxicity but inhibition of proliferation is not observed until higher concentrations are included in the culture system. Inhibition is not observed with supernatants from a breast cancer cell line nor with supernatants from normal cells. There was complete concordance between the capacity of the tumour cells themselves to inhibit LAK generation and the presence of inhibitory activity in the corresponding supernatant. The inhibitory factor(s) is stable after heating to 44 and 56 degrees C. Production of the inhibitory factor(s) is sensitive to metabolic inhibitors and has a molecular weight greater than 25 kD. The inhibition of LAK cell stimulation by tumour cells may partially explain the failure of adoptively transferred LAK cells and IL-2 therapy to cause tumour regression in man.


Br. J. Cancer (1989), 59, 515–521 © The Macmillan Press Ltd., 1989

### Suppression of the generation of lymphokine-activated killer (LAK) cells by serum-free supernatants of *in vitro* maintained tumour cell lines

P.J. Guillou, C.W. Ramsden, S.S. Somers & P.C. Sedman

*University Department of Surgery, St James's University Hospital, Leeds LS9 7TF, UK.*

**Summary** Serum-free supernatants from *in vitro* maintained gastrointestinal cancer and melanoma cell lines inhibit the generation of lymphokine (IL-2) activated killer (LAK) cells in a time and dose-related manner. Concentrations as low as 5% can inhibit the generation of LAK cytotoxicity but inhibition of proliferation is not observed until higher concentrations are included in the culture system. Inhibition is not observed with supernatants from a breast cancer cell line nor with supernatants from normal cells. There was complete concordance between the capacity of the tumour cells themselves to inhibit LAK generation and the presence of inhibitory activity in the corresponding supernatant. The inhibitory factor(s) is stable after heating to 44 and 56°C. Production of the inhibitory factor(s) is sensitive to metabolic inhibitors and has a molecular weight greater than 25 kD. The inhibition of LAK cell stimulation by tumour cells may partially explain the failure of adoptively transferred LAK cells and IL-2 therapy to cause tumour regression in man.

Under certain circumstances lymphocytes derived from the peripheral blood (PBL) or from tumour-infiltrating leukocyte (TIL) populations become highly cytotoxic towards tumour cells on exposure to the T-cell derived lymphokine, interleukin 2 (IL-2) (Grimm *et al.*, 1982; Phillips *et al.*, 1986; Roberts *et al.*, 1987). In the case of PBL the cytotoxicity is non-MHC-restricted and the cytotoxic cell precursors are mainly derived from cells expressing the phenotype of natural killer cells (Ortaldo *et al.*, 1986; Roberts *et al.*, 1987), and are termed lymphokine-activated killer (LAK) cells following exposure to IL-2. In contrast the cytotoxicity exerted by lymphocytes derived from TIL may be MHC-restricted and such cells possess T-cell characteristics (Muul *et al.*, 1987; Whiteside *et al.*, 1988). Adoptive cellular immunotherapy with either syngeneic LAK or TIL cells expanded *in vitro* with IL-2 causes the regression of established metastases in a wide variety of murine tumour models (Eberlein *et al.*, 1982; Lafreniere *et al.*, 1985; Mule *et al.*, 1986*b*; Rosenberg *et al.*, 1986). Unfortunately this experience is not paralleled by the clinical application of such an approach where some 60% of patients with metastatic disease fail to respond to this therapy (Rosenberg *et al.*, 1987; West *et al.*, 1987; Topalian *et al.*, 1988). Similarly, treatment with IL-2 alone appears to be ineffective (Lotze *et al.*, 1986) despite the fact that many human tumours are infiltrated by host mononuclear cells which might become cytotoxic following exposure to IL-2 *in vivo* (Svennevig *et al.*, 1984; Cozzolino *et al.*, 1986).

Prompted by the observations that both IL-2 production and LAK cell generation were impaired with patients with tumours which had extended beyond local confines (Monson *et al.*, 1986, 1987), we have previously shown that the presence of low numbers of some cell-line derived tumour cells was found to potently inhibit LAK cell generation *in vitro* (Guillou *et al.*, 1989). A number of reports have demonstrated that supernatants derived from tumour cell lines can inhibit lymphocyte proliferation and cytokine production (Werkmeister *et al.*, 1980; Meischner *et al.*, 1986; Ebert *et al.*, 1987; Pommier *et al.*, 1987; Farram *et al.*, 1982; Hersey *et al.*, 1983). However, the effects of these supernatants on the generation of cytotoxic responses has rarely been examined (Cozzolino *et al.*, 1987).

The present experiments represent an extension of our previous studies, which have shown that LAK cell generation can be suppressed by tumour cells *in vitro*. Our aim was to determine whether or not such inhibition might be attributable to tumour-derived soluble suppressor factorsand to perform a preliminary physicochemical characterisation of such factors.

### Materials and methods

### *Mononuclear cell separation and culture conditions*

Peripheral blood mononuclear cells were isolated from the heparinised blood of healthy donors by centrifugation on 'lymphoprep' columns (Nyegaard UK Ltd) and depleted of adherent cells by 2 h incubation on plastic Petri dishes (Nunc). The non-adherent cells were suspended in RPMI 1640 medium (Gibco) containing gentamicin ($160\,\mu g\,ml^{-1}$), Fungizone ($2.5\,\mu g\,ml^{-1}$), sodium pyruvate (2 mM), 1% non-essential amino acids, 2-mercaptoethanol (0.05 mM) and 10% mycoplasma-free fetal calf serum. Lymphocyte culture was performed in $75\,cm^2$ upstanding tissue culture flasks containing a total volume of 20 ml and the final lymphocyte density was $1.5\times10^6\,ml^{-1}$. Recombinant IL-2 (Roche) was added to all cultures at a concentration of 1,000 units $ml^{-1}$.

All the experiments described included a control culture to which no putative suppressor element had been added. The flasks were incubated at 37°C for four days in a humidified atmosphere of 5% $CO_2$ in air. At the end of this period the activated effector cells were harvested, counted and corrected to a concentration of $5\times10^6$ viable lymphocytes $ml^{-1}$ in complete medium for use in the LAK cytotoxicity assay as described below.

In some experiments, aliquots of the LAK cells obtained as described above were seeded into the wells of flat-bottomed microplates ($10^5$ cells per well in five replicates each in a volume of 0.1 ml). Each well was pulsed with $1\,\mu Ci$ $^3H$-thymidine and further incubated at 37°C overnight. These proliferating lymphocytes were then harvested on to filter discs and the amount of $^3H$-thymidine incorporated measured in a beta-counter.

### *Tumour cell lines*

Studies of the effects of human tumour cell lines or their supernatants on the generation of LAK cells have been

Correspondence: P.J. Guillou, Academic Surgical Unit, Queen Elizabeth the Queen Mother Wing, St Mary's Hospital Medical School, London W2 1NY, UK.
Received 27 September 1988, and in revised form, 14 November 1988.

performed using the following cell lines. SKBr3 is derived from a human carcinoma of the breast and is maintained in Dulbecco's modified minimum essential medium. HT-29, COLO320 and COLO205 are colorectal cancer cell lines maintained in RPMI 1640 medium, as is the pancreatic cancer cell line MiPaCa. The human malignant melanoma cell line G361 is maintained in McCoy's medium 5A and the human amniotic cell line, WISH, is maintained in Dulbecco's modified MEM. The cell lines were all mycoplasma-free as determined using the Hoechst stain no. 33258. In experiments in which the tumour cells were added directly to the LAK generation cultures, they were obtained from the confluent monolayers after a brief trypsinisation followed by six washes in complete medium, before re-suspension at a cell density appropriate to a final lymphocyte: tumour cell ratio of 50:1, as previously described (Guillou *et al.*, 1989).

*Preparation of tumour cell line supernatants*

Tumour cell monolayers were grown to confluence and the medium was discarded. The flasks were gently washed in serum-free medium so as not to disturb the monolayer but to remove as much FCS as possible, before reculturing for a further 24 h in serum-free RPMI 1640 medium containing all the usual additives. This medium was then recovered, centrifuged to remove any debris and stored at $-70°C$ until studied as a putative suppressor of LAK cell generation. In all the experiments to be described supernatants were obtained from monolayers which were confluent at the time of harvest and appeared healthy at the end of the 24 h culture period in serum-free medium. Cultures and supernatants in which the tumour cells had detached from their monolayer were not used.

In some experiments the effects of metabolic inhibitors on the release of suppressor factors into the supernatants was studied. Tumour cells were grown to within 48 h of confluence and the medium replaced with complete medium containing various concentrations of mitomycin C, actinomycin D or cycloheximide (all obtained from Sigma Chemicals UK). The monolayers were cultured for a further hour in the presence of these metabolic inhibitors and then this medium was removed, the cells gently washed and re-covered with fresh serum-free medium which was harvested and stored a further 24 h later as described above. The final concentrations of these inhibitors were selected such that they did not cause gross tumour cell detachment and the cells were again confluent at the time of supernatant harvesting. Optimum results were obtained with $0.1 \mu g ml^{-1}$ of mitomycin C, $0.05 \mu g ml^{-1}$ of actinomycin D and between 1 and $10 \mu g ml^{-1}$ of cycloheximide. That the inhibitors were preventing DNA, RNA and protein synthesis was confirmed in microplate tumour cell monolayers treated identically to the bulk tumour cell cultures and then pulsed for 12 h with tritiated thymidine, uridine or leucine respectively. The isotope incorporation into these cultures was diminished but not eliminated in comparison with that of identical untreated cultures of the same tumour cell lines.

*Temperature sensitivity of suppressive supernatants*

Tumour cell line supernatants with suppressive effects on LAK cell generation were heated at 44, 56 and 70°C for 30 min before their addition to LAK generation cultures.

*Amicon filtration*

Suppressive supernatant samples were enclosed in Amicon microconcentrator filters of pore diameters corresponding to molecular weights of 15, 25 and 125 kD for 2 h. The concentrates were then reconstituted to their original volumes with RPMI 1640 medium before their effects on LAK cell generation were studied.

*Cytotoxicity assay*

This was a standard 4 h $^{51}Cr$ release assay as previously described (Monson *et al.*, 1987). Doubling dilutions of $5 \times 10^5$ effector cells in 0.1 ml complete medium were added to $10^4$ $^{51}Cr$-labelled target cells in 0.1 ml complete medium in triplicate round-bottomed microtitre wells. After 4 h 100 $\mu l$ of the supernatant was harvested from each well and the radioactivity counted in a gamma-counter. The percentage specific $^{51}Cr$ release at each effector: target cell ratio was calculated as previously described. Data are expressed as percentage specific $^{51}Cr$ release at the stated effector: target cell ratio and also, where appropriate, as lytic units per $10^7$ effector cells. Percentage suppression was calculated on the basis of the lytic unit figures.

### Results

*Concordance between tumour cell lines and their supernatants as suppressors of LAK cell generation* in vitro

Table I summarises the results of a series of experiments whose objective was to identify whether or not the supernatants from tumour cell lines were capable of impairing LAK cell generation *in vitro*. LAK cells were generated either alone or in the presence of tumour cells (at a ratio of 1 per 50 lymphocytes) or their supernatants at a final concentration of 20% in culture. These data confirm our previous report that the presence of tumour cells derived from certain cell lines (G361, HT-29, COLO205, COLO320, MiPaCa), but not others (SKBr3 and the non-malignant cell line WISH), can suppress the generation of LAK cells *in vitro*. Moreover, the presence of 20% serum-free supernatant from the suppressive cell lines was also highly suppressive of LAK cell generation whereas no suppression was seen with the supernatants of the non-suppressive cell lines. Similarly, when supernatants from non-malignant cells (24 h cultures of allogeneic endothelial cells obtained from human umbilical cord as previously described (Guillou *et al.*, 1989)) were added at concentrations as high as 20% or more, no suppression of LAK cell generation was observed. Thus, so far, we have found concordance between the LAK cell suppression seen with the tumour cells and that caused by the presence of their respective supernatants.

*Dose and time-related effects of tumour cell supernatants on LAK cell generation and proliferation*

Figure 1 represents the results of one representative experiment in which different concentrations of the tumour cell supernatant were present in the LAK generation cultures. It can be seen that concentrations of the COLO205 supernatant as low as 5% inhibited LAK cell generation and almost complete inhibition was observed with concentrations of 10 or 20%. Similar results were obtained with all the other suppressive cell line supernatants, maximum suppression being obtained with concentrations of 20%. In contrast, concentrations of supernatants from the non-suppressive cell lines had no effect on LAK cell generation at concentrations as high as 50% of the total culture volume. Figure 2 shows the effects of the COLO205 supernatant on the proliferation of these same IL-2 activated lymphocytes as assessed by the uptake of $^3H$-thymidine. Inhibition of lymphocyte proliferation was significantly diminished only at supernatant concentrations of 10% or more.

In Figure 3 it can be seen that the inhibitory effects of the tumour cell line supernatants were also temporally related. Significant inhibition of LAK generation occurred only when the supernatant was present within the first 48 h of culture, there being no suppression when the addition of the supernatant was delayed until the third or fourth days of culture.

*Effects of tumour cell supernatants on cytotoxic effector cell function* in vitro

The data shown in Figure 3 suggested that suppressive tumour cell supernatants have no effect on LAK cytotoxicity

**Table I** Effects of tumour cell lines and their supernatants on LAK cell generation

| | | Percentage cytotoxicity | | | |
|---|---|---|---|---|---|
| *Effector cell* | *Target* | *50:1* | *12:1* | *LU* | *% Suppression* |
| *Exp. 1* | | | | | |
| LAK alone | G361 | 41.3 | 15.7 | 34.7 | – |
| LAK/G361 cells | G361 | 14.6 | 6.4 | 4.3 | 87.6 |
| LAK/20%G361Sup[a] | G361 | 4.2 | 0.3 | 0.005 | 99.9 |
| LAK/SKBr3 cells | G361 | 40.8 | 15.0 | 34.0 | 2.0 |
| LAK/20%SKBr3Sup[a] | G361 | 38.5 | 17.4 | 28.0 | 19.3 |
| LAK/HT29 cells | G361 | 9.3 | 2.8 | 0.002 | 99.9 |
| LAK/20%HT29Sup[a] | G361 | 5.3 | 3.0 | 0.001 | 99.9 |
| *Exp. 2* | | | | | |
| LAK alone | COLO320 | 35.4 | 19.3 | 25.3 | – |
| LAK/COLO320 cells | COLO320 | 23.2 | 9.9 | 5.9 | 76.7 |
| LAK/20%COLO320 Sup[a] | COLO320 | 25.6 | 11.5 | 9.6 | 62.1 |
| LAK/SKBr3 cells | COLO320 | 36.4 | 19.0 | 24.3 | 3.9 |
| LAK/20%SKBrSup[a] | COLO320 | 35.8 | 18.0 | 22.8 | 9.9 |
| *Exp. 3* | | | | | |
| LAK alone | G361 | 84.7 | 49.1 | 163.0 | – |
| LAK/G361 cells | G361 | 20.6 | 6.1 | 2.0 | 98.8 |
| LAK/20%G361Sup[a] | G361 | 13.3 | 4.0 | 0.23 | 99.9 |
| LAK/SKBr3 cells | G361 | 85.6 | 49.1 | 137.1 | 15.9 |
| LAK/20%SKBr3Sup[a] | G361 | 81.7 | 43.3 | 136.3 | 16.4 |
| LAK/MiPaCa cells | G361 | 28.6 | 10.3 | 9.6 | 94.1 |
| LAK/20%MiPaCaSup[a] | G361 | 48.9 | 6.9 | 12.8 | 92.1 |
| *Exp. 4* | | | | | |
| LAK alone | SKBr3 | 45.8 | 25.9 | 52.0 | – |
| LAK/G361 cells | SKBr3 | 16.4 | 8.4 | 0.7 | 98.7 |
| LAK/20%G361Sup[a] | SKBr3 | 11.4 | 6.7 | 0.03 | 99.9 |
| LAK/WISH cells | SKBr3 | 47.7 | 24.7 | 52.1 | 0.2 |
| LAK/20%WISHSup[a] | SKBr3 | 53.9 | 31.8 | 72.8 | −40.0 |
| *Exp. 5* | | | | | |
| LAK alone | SKBr3 | 75.5 | 35.7 | 86.3 | – |
| LAK/COLO205 cells | SKBr3 | 27.7 | 8.8 | 11.01 | 87.2 |
| LAK/20%COLO205Sup[a] | SKBr3 | 7.9 | 0.9 | 0.004 | 99.99 |
| LAK/SKBr3 cells | SKBr3 | 74.8 | 39.5 | 99.3 | −15.1 |
| LAK/20%SKBr3Sup[a] | SKBr3 | 73.8 | 34.3 | 88.9 | −3.0 |
| *Exp. 6* | | | | | |
| LAK alone | SKBr3 | 24.8 | 7.1 | 8.3 | – |
| LAK/20%HT29Sup[a] | SKBr3 | 1.9 | 0.4 | 0.001 | 99.9 |
| LAK/20%Endothelial cell Sup[a] | SKBr3 | 25.01 | 6.0 | 8.8 | −5.0 |
| *Exp. 7* | | | | | |
| LAK alone | COLO320 | 45.8 | 25.9 | 52 | – |
| LAK/20%G361Sup[a] | COLO320 | 11.4 | 6.7 | 0.03 | 99.9 |
| LAK/20%Endothelial cell Sup[a] | COLO320 | 47.7 | 25.7 | 52.1 | −0.2 |

[a]Tumour cell and endothelial cell supernatants were serum-free and collected after 24 h of cell culture. These were added to LAK cell generation cultures at a final concentration of 20%. Tumour cells were added to LAK generation cultures at a ratio of one tumour cell per 50 lymphocytes. LAK cytotoxicity was measured 4 days later and the data are expressed as percentage specific $^{51}Cr$ release from the indicated target cell at the effector:target cell ratios shown. Lytic units were calculated from data obtained at all the effector:target cell ratios and percentage suppression calculated by comparison with the lytic units measured in the corresponding control cultures containing no putative suppressor element (designated 'LAK alone').

when added to the LAK generation cultures late in the culture period and thus do not directly impair the binding, lethal hit or lytic phases of cellular cytotoxicity. In order to confirm this we conducted experiments in which tumour cell supernatants were incorporated into the cytotoxicity assay directly and these data are shown in Table II. It can be seen that even at concentrations as high as 40% little inhibition of LAK cell cytotoxicity was caused by the presence of the tumour cell supernatants.

*Temperature sensitivity of the suppressive supernatants*

The suppression observed was insensitive to heating at 44 or 56°C for 30 min but was eliminated by heating to 70°C for the same time period (Table III).

*Effects of Amicon filtration on the suppression of LAK cell generation*

Table IV summarises the results of several experiments in which the suppressive supernatants were subjected to Amicon filtration, reconstituted to their original volumes and incorporated in the LAK generation cultures at a final concentration of 20%. Loss of suppressive activity was seen only when the supernatant was subjected to filtration through a microconcentrater of pore size corresponding to a

**Figure 1** Effects of different concentrations of serum-free supernatant from COLO 205 colorectal cancer cell line on the generation of lymphokine-activated killer cells *in vitro* (mean percentage specific $^{51}Cr$ release $\pm$s.d.).

**Figure 3** Relationship between the suppression of LAK cell generation by G361 melanoma cell line supernatant and time of addition of the supernatant. For reasons of clarity, the data for cultures to which no supernatant was added are omitted, but the cytotoxicity was identical to that of cultures to which supernatants were added on the day of assay, i.e. day 4.

**Figure 2** Effects of COLO 205 supernatant on IL-2 stimulated $^{3}H$-thymidine uptake.

**Table II** Effects of tumour cell supernatants on the cytotoxicity assay *in vitro*

| | *Conc. supernate in $^{51}Cr$-release assay* | | | |
|---|---|---|---|---|
| *Source of supernatant* | *5%* | *10%* | *20%* | *40%* |
| Fresh RPMI 1640 | 29.0[a] | 29.0 | 27.5 | 25.3 |
| COLO320 | 27.0 | 25.1 | 22.4 | 19.9 |
| HT-29 | 27.0 | 25.1 | 24.7 | 21.1 |
| SKBr3 | 26.9 | 25.2 | 23.6 | 22.7 |
| COLO205 | 27.3 | 27.4 | 25.7 | 26.5 |
| G361 | 29.5 | 27.5 | 24.7 | 21.1 |

LAK cells were generated from PBMC in the standard manner and then tested in the $^{51}Cr$-release assay at an effector:target cell ratio of 50:1. Serum-free medium from each of the tumour cell lines described was added directly to the $^{51}Cr$-release assay at the concentrations indicated; [a]Figures refer to the percentage specific $^{51}Cr$-release from $^{51}Cr$-labelled SKBr3 target cells at a single effector:target ratio of 50:1. Specific $^{51}Cr$-release at 50:1 in the absence of supernatant was 29.0%.

**Table III** Thermosensitivity of tumour-derived suppressor factor

| | | *% Cytotoxicity* | | | |
|---|---|---|---|---|---|
| *Temperature* | *Target* | *50:1* | *12:1* | *LU* | *% Suppression* |
| Control (LAK alone) | SKBr3 | 32.5 | 12.4 | 17.8 | – |
| Untreated supernate | SKBr3 | 1.7 | 1.4 | <0.001 | 99.99 |
| 44°C for 30 min | SKBr3 | 1.8 | 0.03 | <0.001 | 99.99 |
| 56°C for 30 min | SKBr3 | 1.71 | 0.4 | <0.001 | 99.99 |
| 70°C for 30 min | SKBr3 | 30.3 | 11.8 | 14.7 | 17.4 |

Serum-free supernatant from G361 melanoma was heated to the temperatures recorded and added to LAK generation cultures at a final concentration of 20%. LAK cytotoxicity was measured against SKBr3 cells after four days in culture and is expressed as % specific $^{51}Cr$ release at the effector: target cell ratios shown as well as lytic units. Percentage suppression was calculated as described in the legend to Table I.

**Table IV** Effects of Amicon filtration on tumour-derived suppressor factor

| | | *% Cytotoxicity* | | | |
|---|---|---|---|---|---|
| *Filter pore diameter* | *Target* | *50:1* | *12:1* | *LU* | *% Suppression* |
| Control (LAK alone) | SKBr3 | 67.8 | 34.6 | 85.4 | – |
| Unfiltered supernate | SKBr3 | 5.4 | 0.5 | 0.01 | 99.99 |
| 15 kD filter | SKBr3 | 2.8 | 0.3 | 0.01 | 99.99 |
| 25 kD filter | SKBr3 | 7.7 | 1.0 | 0.1 | 99.9 |
| 125 kD filter | SKBr3 | 69.5 | 35.0 | 86.8 | –1.6 |

Serum-free G361 supernatant was subjected to Amicon filtration with filters of differing pore diameters. The residual supernatant was reconstituted to the original volume with RPMI 1640 medium and added to LAK generation cultures at a concentration of 20%. LAK cytotoxicity was measured 4 days later and the data expressed as described in Table I.

**Figure 4** Effects of metabolic inhibitors on the suppressive effects of tumour cell line supernatants on LAK cell generation.

molecular weight of 125 kD, there being no loss of activity with filters of 15 or 25 kD porosity.

*Effects of metabolic inhibitors on the production of suppressor factors in tumour cell supernatants*

The inhibition of protein synthesis by cycloheximide was markedly inhibitory towards the production of the suppressor factor (Figure 4). The suppression caused by the tumour cell line-derived supernatants was almost completely inhibited by treatment of the tumour cells with 10 $\mu$g ml$^{-1}$ of cycloheximide. In addition the inhibition of DNA and RNA synthesis (as assessed by the uptake of radiolabelled thymidine and uridine respectively by the tumour cells) was also effective in impairing the production of the LAK cell suppressor factors present in the supernatants derived from confluent tumour cell lines.

### Discussion

The administration of IL-2 activated lymphocytes as therapeutic agents for human cancer currently meets with only limited success and cannot compare with the results accomplished with the same approach in experimental animals. However, in making this comparison differences between the murine and human application of adoptive cellular immunotherapy require consideration. In the murine studies activated lymphocytes are derived from normal, non-tumour-bearing syngeneic animals, whereas in clinical application the activated cells are of course autologous to the patient. If tumour-mediated suppression of lymphocyte activation does occur *in vivo*, the cytolytic efficacy of autologous lymphocytes might be less than that of lymphocytes derived from healthy individuals, and we have already shown this to be the case (Monson *et al*., 1986, 1987). Furthermore, both clinically and experimentally, the administration of IL-2 alone (without adoptive cellular immunotherapy) fails to accomplish tumour regression suggesting that the suppressive mechanisms operative in man also exist in murine models (Mule *et al*., 1986*a*; Salup *et al*., 1986; Rosenberg *et al*., 1987). We have attempted to investigate the role of the tumour cell (as opposed to host factors such as suppressor cells) in this context.

The experiments reported in this paper confirm and extend our previous report that cells derived from some continuously growing tumour cell lines impair the generation of lymphokine-activated killer cells *in vitro*. Thus cells derived from cultures of human malignant melanoma, colorectal and pancreatic cancer, but not from the breast cancer cell line SKBr3 nor from WISH cells, can inhibit LAK cell generation *in vitro*. Moreover, in the present investigations we have found that serum-free supernatants from the suppressive, but not from the non-suppressive, cell lines can also potently inhibit LAK cell generation in a dose and time-related manner. The concordance between the suppression caused by the tumour cells and that caused by their corresponding supernatants suggests that the inhibition is mediated via the release of a soluble factor(s) from the tumour cells. The time course of the inhibition obtained with the supernatants is identical with that we have previously reported for the inhibition of LAK cell function by the tumour cells themselves, i.e. that the putative suppressor factor(s) must be present within the first 48 h of the initiation of culture (Guillou *et al*., 1988). Further studies are required to determine whether other cell lines of non-malignant or malignant origin cause similar effects.

The suppressor factor(s) does not directly inhibit cytotoxicity at the level of the effector cell as evidenced by the data shown in Table II. Furthermore in experiments not shown here, we have been unable to render the tumour target cells resistant to cytotoxicity by prior culture in the supernatant. Thus the tumour-derived factors do not appear to work in a manner analogous to that of the protective effects which interferons are able to exert on natural killer and LAK cell tumour targets (De Fries *et al*., 1988; Yeoman *et al*., 1988).

That prior treatment of the tumour cells with inhibitors of DNA, RNA and protein synthesis eliminates the inhibition of LAK cell induction observed in the presence of the tumour cell supernatants suggests that the suppressor factors are proteins coded for in the tumour genome. However, interference by virally encoded proteins cannot be excluded. Indeed, one such viral peptide, the retroviral envelope protein P15E, has been implicated in the suppression of lymphocyte proliferation (Snyderman *et al*., 1984). P15E perturbs monocyte function and directly inhibits T-lymphocyte proliferation (Harrell *et al*., 1986; Copelan *et al*., 1983). Although P15E is normally only found in the envelope of lymphotropic or leukaemic viruses this peptide has been detected in murine B16 melanoma and is also expressed in lectin-activated lymphoblasts (Cianciolo *et al*., 1983, 1984). It has been shown that monoclonal antibodies to P15E can prevent the suppressive effects of certain bovine tumour extracts on delayed hypersensitivity *in vivo* (Nelson *et al*., 1985). As observed with the tumour supernatants reported herein, the suppressive effects of P15E are resistant to heating at 56°C. In contrast, our preliminary crude estimate of the molecular weight of the factor(s) present in our supernatants indicate something in excess of 25 kD rather than the 15 kD of P15E. More directly, however, although a synthetic peptide homologous to P15E inhibits both spontaneous and interferon-boosted human NK activity, it has not been found to impair the activation of NK cells by IL-2 (Harris *et al*., 1987).

A number of alternative candidate molecules for the supernatant suppressor factor described in our studies exist in the literature. Putnam & Roth (1985) have described a heat-stable, acid-labile glycoprotein derived from murine melanoma which can inhibit proliferative responses to lectins and IL-2. However, the molecular weight of this factor was only of the order of 10–12 kD but was distinct from P15E. Pommier *et al*. (1987) and Ebert *et al*. (1987) have described a factor present in conditioned medium from HT-29 cells which was able to inhibit IL-2 production and lymphocyte proliferation and whose effects could not be prevented by the addition of further IL-2. The molecular weight of their factor was around 56 kD and because our factor(s) also appear to inhibit lymphocyte proliferation at higher concentrations the two may be identical. In our experiments the induction of LAK cell cytotoxicity was impaired at lower concentrations of supernatant than those required to inhibit proliferation, in keeping with previous suggestions that the *in vitro* induction of LAK cells is not dependent on DNA synthesis (Malkovsky *et al*., 1987). This antiproliferative effect may have some *in vivo* significance, however, since experimentally IL-2 stimulates the proliferation of adoptively transferred LAK cells, the proliferation apparently being essential for therapeutic efficacy (Ettinghausen *et al*., 1985).

Mieschner *et al.* (1986) have also reported that gastrointestinal cancer cells and their products can also repress the proliferation of tumour infiltrating lymphocytes. Tumour cell lines derived from a variety of colorectal carcinomas appear to secrete both alpha and beta transforming growth factors (Coffey *et al.*, 1986; Hanauske *et al.*, 1987), the latter having recently been described as a potent inhibitor of NK cell activity and murine LAK cell induction (Rook *et al.*, 1986). These growth factors might therefore represent potential candidates for the inhibitory substances present in our tumour cell supernatants.

Whiteside *et al.* (1988) have experienced difficulty in generating proliferating lymphocytes from TIL extracted from metastatic head and neck and colorectal cancers. Even when so generated (following a lag period of some 30–40 days) such proliferating lymphocytes exert little or no cytolytic activity, suggesting that the suppressive effects seen in our *in vitro* experiments may also be operative *in vivo*. It is evident that caution must be exercised in extrapolating the results of these *in vitro* experiments to clinical *in vivo* circumstances. Nevertheless, it is tempting to speculate that the experiments we have described may lead to at least a partial explanation of the failure of IL-2 therapy to cause durable remission in patients suffering from advanced malignant disease. The relationship of the factors which we have observed to those described by others remains to be elucidated and we are currently attempting to characterise them in greater detail. However, if these studies succeed in demonstrating that such factors have *in vivo* relevance then their characterisation may result in the development of further strategies aimed at augmenting the clinical response rates to immunotherapy.

### References

CIANCIOLO, G.J., LOSTROM, M.E., TAM, M. & SNYDERMAN, R. (1983). Murine malignant cells synthesize a 19,000 dalton protein which is physicochemically and antigenically related to the immunosuppressive retroviral protein, p15E. *J. Exp. Med.*, **158**, 885.

CIANCIOLO, G.J., PHIPPS, D. & SNYDERMAN, R. (1984). Human malignant and mitogen transformed cells contain retroviral p15E related antigen. *J. Exp. Med.*, **159**, 964.

COFFEY, R.J., SHIPLEY, G.D. & MOSES, H.L. (1986). Production of transforming growth factors by human colon cancer cell lines. *Cancer Res.*, **46**, 1164.

COPELAN, E.A., RINEHART, J.J., LEWIS, M., MATHES, L., OLSEN, R. & SAGONE, A. (1983). The mechanism of retrovirus suppression of human T cell proliferation *in vitro*. *J. Immunol.*, **131**, 2017.

COZZOLINO, F., TORCIA, M., CASTIGLI, E. & 7 others (1986). Presence of activated T-cells with a $T8^+$, $M1^+$, $Leu7^+$ surface phenotype in invaded lymph nodes from patients with solid tumours. *JNCI*, **77**, 637.

COZZOLINO, F., TORCIA, M., CAROSSINO, A.M. & 7 others (1987). Characterization of cells from invaded lymph nodes in patients with solid tumours. Lymphokine requirement for tumour-specific lymphoproliferative response. *J. Exp. Med.*, **166**, 303.

DE FRIES, R.U. & GOLUB, S.H. (1988). Characteristics and mechanism of IFN-gamma-induced protection of human tumor cells from lysis by lymphokine-activated killer cells. *J. Immunol.*, **140**, 3686.

EBERLEIN, T.J., ROSENSTEIN, M.R. & ROSENBERG, S.A. (1982). Regression of a syngeneic solid tumor by systemic transfer of lymphoid cells expanded in interleukin 2 in mice. *J. Exp. Med.*, **156**, 385.

EBERT, E.C., ROBERTS, A.I., O'CONNELL, S.M., ROBERTSON, F.M. & NAGASE, H. (1987). Characterization of an immunosuppressive factor derived from human colon cancer cells. *J. Immunol.*, **138**, 2161.

ETTINGHAUSEN, S.E., LIPFORD, E.H., MULE, J.J. & ROSENBERG, S.A. (1985). Recombinant interleukin 2 stimulates *in vivo* proliferation of adoptively transferred lymphokine-activated killer cells. *J. Immunol.*, **135**, 3899.

FARRAM, E., NELSON, M., NELSON, D.S. & MOON, D.K. (1982). Inhibition of cytokine production by a tumor cell product. *Immunology*, **46**, 603.

GRIMM, E.A., MAZUMDER, A., ZHANG, H.Z. & ROSENBERG, S.A. (1982). Lymphokine-activated killer cell phenomenon. Lysis of natural killer-resistant fresh solid tumor cells by interleukin 2-activated autologous human peripheral blood lymphocytes. *J. Exp. Med.*, **155**, 1823.

GUILLOU, P.J., SEDMAN, P.C. & RAMSDEN, C.W. (1989). The inhibition of lymphokine-activated killer cell generation by cultured tumour cell lines *in vitro*. *Cancer Immunol. Immunother.*, **28**, 43.

HANAUSKE, A.R., BUCHOK, J., SCHEITHAUER, W. & VON HOFF, D.D. (1987). Human colon cancer cells lines secrete alpha-TGF-like activity. *Br. J. Cancer*, **55**, 57.

HARRELL, R.A., CIANCIOLO, G.J., COPELAND, T.D., OROSZLAN, S. & SNYDERMAN, R. (1986). Suppression of the respiratory burst of human monocytes by a synthetic peptide homologous to the envelope proteins of human and animal retroviruses. *J. Immunol.*, **136**, 3517.

HARRIS, D.T., CIANCIOLO, G.J., SNYDERMAN, R., ARGOV, S. & KOREN, H.S. (1987). Inhibition of human natural killer cell activity by a synthetic peptide homologous to a conserved region in the retroviral protein p15E. *J. Immunol.*, **138**, 889.

HERSEY, P., BINDON, C., CZERIECKI, M., SPURLING, M., WASS, J. & MCCARTHY, W.H. (1983). Inhibition of interleukin 2 production by factors released from tumor cells. *J. Immunol.*, **131**, 2837.

LAFRENIERE, R. & ROSENBERG, S.A. (1985). Successful immunotherapy of murine experimental hepatic metastases with lymphokine-activated killer cells and recombinant interleukin 2. *Cancer Res.*, **45**, 3735.

LOTZE, M.T., MATORY, Y.L., RAYNER, A.A. & 4 others (1986). Clinical effects and toxicity of interleukin-2 in patients with cancer. *Cancer*, **58**, 2772.

MALKOVSKY, M., JIRA, M., MADAR, J., MALKOVSKA, V., LOVELAND, V. & ASHERSON, G.L. (1987). Generation of lymphokine-activated killer cells does not require DNA synthesis. *Immunology*, **60**, 471.

MIESCHNER, S., WHITESIDE, T.L., CAREEL, S. & VON FLIEDNER, V. (1986). Functional properties of tumor-infiltrating and blood lymphocytes in patients with solid tumours: effects of tumour cells and their supernatants on proliferative responses of lymphocytes. *J. Immunol.*, **136**, 1899.

MONSON, J.R.T., RAMSDEN, C.W. & GUILLOU, P.J. (1986). Decreased interleukin-2 production in patients with gastrointestinal cancer. *Br. J. Surg.*, **73**, 483.

MONSON, J.R.T., RAMSDEN, C.W., GILES, G.R., BRENNAN, T.G. & GUILLOU, P.J. (1987). Lymphokine-activated killer (LAK) cells in patients with gastrointestinal cancer. *Gut*, **28**, 1420.

MULE, J.J., ETTINGHAUSEN, S.E., SPIESS, P.J. & ROSENBERG, S.A. (1986*a*). Antitumor efficacy of lymphokine-activated killer cells and interleukin 2 *in vivo*: survival benefit and mechanisms of tumor escape in mice undergoing immunotherapy. *Cancer Res.*, **46**, 676.

MULE, J.J., YANG, J., SHU, S. & ROSENBERG, S.A. (1986*b*). The antitumor efficacy of lymphokine-activated killer cells and recombinant interleukin 2 *in vivo*: direct correlation between reduction of established metastases and cytolytic activity of lymphokine-activated killer cells. *J. Immunol.*, **136**, 3899.

MUUL, L.M., SPIESS, P.J., DIRECTOR, E.P. & ROSENBERG, S.A. (1987). Identification of specific cytolytic immune responses against autologous tumor in humans bearing malignant melanoma. *J. Immunol.*, **138**, 989.

NELSON, M., NELSON, D.S., SPRADBROW, P.B. & 4 others (1985). Successful tumour immunotherapy: possible role of antibodies to anti-inflammatory factors produced by neoplasms. *Clin. Exp. Immunol.*, **61**, 109.

ORTALDO, J.R., MASON, A. & OVERTON, R. (1986). Lymphokine-activated killer cells. Analysis of progenitors and effectors. *J. Exp. Med.*, **164**, 1193.

PHILLIPS, J.H. & LANIER, L.L. (1986). Dissection of the lymphokine-activated killer cell phenomenon. Relative contribution of peripheral blood natural killer cells, and T lymphocytes to cytolysis. *J. Exp. Med.*, **164**, 814.

POMMIER, G.J., GARROUSTE, F.L., BETTETINI, D., CULOUSCOU, J.M. & REMACLE-BONNET, M.M. (1987). *In vivo* delayed rejection of tumors and inhibition of delayed-type hypersensitivity by HT-29 human colonic adenocarcinoma cell line. *Cancer Immunol. Immunother.*, **24**, 225.

PUTNAM, J.B. & ROTH, J.A. (1985). Identification and characterization of a tumor-derived immunosuppressive glycoprotein from murine melanoma K-1735. *Cancer Immunol. Immunother.*, **19**, 90.

ROBERTS, K., LOTZE, M.T. & ROSENBERG, S.A. (1987). Separation and functional studies of the human lymphokine-activated killer cell. *Cancer Res.*, **47**, 4366.

ROOK, A.H., KEHRL, J.H., WAKEFIELD, L.M. & 5 others (1986). Effects of transforming growth factor B on the functions of natural killer cells: depressed cytolytic activity and blunting of interferon responsiveness. *J. Immunol.*, **136**, 3916.

ROSENBERG, S.A., SPIESS, P. & LAFRENIERE, R. (1986). A new approach to the adoptive immunotherapy of cancer with tumor-infiltrating lymphocytes. *Science*, **233**, 1318.

ROSENBERG, S.A., LOTZE, M.T., MUUL, L.M. & 10 others (1987). A progress report on the treatment of 157 patients with advanced cancer using lymphokine-activated killer cells and interleukin-2 or high dose interleukin-2 alone. *N. Engl. J. Med.*, **316**, 889.

SALUP, R.R. & WILTROUTR, H. (1986). Adjuvant immunotherapy of established murine renal cancer by interleukin 2-stimulated cytotoxic lymphocytes. *Cancer Res.*, **46**, 3358.

SNYDERMAN, R. & CIANCIOLO, G.J. (1984). Immunosuppressive activity of the retroviral envelope protein p15E and its possible relationship to neoplasia. *Immunol. Today*, **5**, 240.

SVENNEVIG, J.L., LUNDE, O.C., HOLTER, J. & BJORGSVIK, D. (1984). Lymphoid infiltration and prognosis in colorectal carcinoma. *Br. J. Cancer*, **49**, 375.

TOPALIAN, S.L., SOLOMON, D., AVIS, F.P. & 9 others (1988). Immunotherapy of patients with advanced cancer using tumor-infiltrating lymphocytes and recombinant interleukin-2: a pilot study. *J. Clin. Oncol.*, **6**, 839.

WERKMEISTER, J., ZAUNDERS, W., McCARTHY, W. & HERSEY, P. (1980). Characterization of an inhibitor of cell division released in tumour cell cultures. *Clin. Exp. Immunol.*, **41**, 487.

WEST, W.H., TAUER, K.H., YANNELLI, J.R.. & 4 others (1987). Constant-infusion recombinant interleukin-2 in adoptive immunotherapy of advanced cancer. *N. Engl. J. Med.*, **316**, 898.

WHITESIDE, T.L., HEO, D.S., TAKAGI, S., JOHNSON, J.T., IWATSUKI, S. & HERBERMAN, R.B. (1988). Cytolytic antitumor effector cells in long term cultures of human tumor-infiltrating lymphocytes in recombinant interleukin 2. *Cancer Immunol. Immunother.*, **26**, 1.

YEOMAN, H. & ROBINS, R.A. (1988). The effect of interferon-gamma treatment of rat tumor cells on their susceptibility to natural killer cell, macrophage and cytotoxic T-cell killing. *Immunology*, **63**, 291.